# COVID-19 in pregnancy: placental pathological patterns and effect on perinatal outcome in five cases

**DOI:** 10.1186/s13000-021-01148-6

**Published:** 2021-10-03

**Authors:** Giovanna Giordano, Chiara Petrolini, Emilia Corradini, Nicoletta Campanini, Susanna Esposito, Serafina Perrone

**Affiliations:** 1 Department of Medicine and Surgery, Pathology Unit, University of Parma, Viale A. Gramsci, 14, 43126 Parma, Italy; 2 Department of Medicine and Surgery, Neonatology Clinic, Pietro Barilla Children’s Hospital, University of Parma, Parma, Italy; 3 Department of Medicine and Surgery, Paediatric Clinic Pietro Barilla Children’s Hospital, University of Parma, Parma, Italy

**Keywords:** Coronavirus disease 2019, Acute respiratory disease, Maternal vascular underperfusion, Foetal vascular underperfusion, Ultrastructural analysis

## Abstract

**Introduction:**

COVID-19, the disease caused by the novel coronavirus SARS-CoV-2, is a severe systemic thrombotic syndrome that emerged in 2019, with an ensuing pandemic.

To evaluate the impact of this disease on placental tissue and perinatal outcome, histological, immunohistochemical and ultrastructural analyses of placental tissue were performed for five cases of pregnant women with COVID-19.

**Case reports:**

All five pregnant women in this series developed COVID-19 in late pregnancy. Two patients experienced respiratory distress, and computed tomography revealed signs of pneumonia, with bilateral involvement, multiple lobular and subsegmental areas of consolidation and ground-glass opacities.

Histological studies of placental tissue revealed the presence of slight signs of maternal vascular underperfusion (MVUs) or foetal vascular underperfusion (FVUs) lesions and mild inflammatory lesions. CD15 immunoreactivity in the placental tissue was low in all cases, demonstrating that in these cases there was not severe foetal hypoxia/asphyxia risk for newborns or distal vascular immaturity.

In all cases examined, ultrastructural analyses showed spherical-like coronavirus particles with an electron intermediate-density core as well as projections from the surface as spike-like structures in the syncytiotrophoblasts. At term, all of the women delivered newborns who were negative for SARS-CoV-2 by nasopharyngeal testing in their first day of life. All newborns were exclusively breastfed and were discharged on the 3rd day of life.

**Conclusions:**

In conclusion, placental patterns in pregnancy due to COVID-19 in the late stage of gestation indicate no evidence of vertical trans-placental SARS-CoV-2 transmission or a significant impact on the perinatal outcome of newborns, in both mild and more severe cases.

## Introduction

COVID-19, that is, coronavirus disease 2019, also known as acute respiratory disease from SARS-CoV-2 or coronavirus disease 2019, is an infectious respiratory disease caused by the virus SARS-CoV-2 belonging to the coronavirus family [1].

The first cases of this disease were found in China in the city of Wuhan in 2019 [1, 2]. The infection spread rapidly throughout China and to adjacent countries as well as other countries. As a result, at a meeting of the WHO Emergency Committee on 30 January, the novel coronavirus 2019 epidemic was declared a Public Health Emergency of International Concern (PHEIC) [3].

The virus responsible for this disease, which was initially isolated from bronchoalveolar lavage fluid samples [2], is a type of single-stranded RNA virus that belongs to the coronavirus family [4]. The known transmission pathways of SARS-CoV-2 in humans include inhalation of tiny droplets carrying the virus, close contact with virus carriers, contact with a surface contaminated by SARS-CoV-2, and aerosol transmission [5].

The virus primarily affects the lower respiratory tract and causes a number of symptoms described as “flu-like”, [6] including fever, cough, shortness of breath, muscle pain, fatigue and gastrointestinal disturbances such as vomiting and diarrhoea [6]. In severe cases, this disease causes pneumonia, acute respiratory distress syndrome, sepsis, septic shock and a cytokine storm with multiorgan failure and, occasionally, disseminated coagulopathy, leading to death [6].

In severe cases, thoracic computed tomography images show pneumonia that exhibits bilateral involvement, with multiple lobular and subsegmental areas of consolidation and ground-glass opacities [6].

Vivanti A et al. reported a case of transplacental transmission of SARS-CoV-2 from a pregnant woman with COVID-19 during late pregnancy. The authors suggested that SARS-CoV-2 can cause the following: (1) maternal viraemia; (2) placental infection, as demonstrated by immunohistochemistry and extremely high viral load, and placental inflammation, as shown by histological examination and immunohistochemistry; and (3) neonatal viraemia following placental infection [7].

Here, we report on a series of cases of pregnant women affected by COVID-19 during late pregnancy who were convalescent with a negative test at the time of delivery. Histological, immunohistochemical, and ultrastructural analyses of the placenta were performed to detect patterns of inflammation and underperfusion lesions and to determine the presence of SARS-CoV-2. Placental features were associated with the perinatal outcome of the offspring.

## Diagnostic assessment

Our case series included five pregnant women with documented COVID-19 in late pregnancy who delivered at the University Hospital of Parma (Italy). Newborn and maternal clinical data were reviewed retrospectively.

The placenta was fixed in 10% buffered formalin and examined by macroscopic and histological analyses. Sections were taken from each placenta for evaluation of the maternal and foetal surfaces, full-thickness membranes and umbilical cord and every evident macroscopic lesion. The sections were stained with haematoxylin and eosin (H&E) after routine processing. The histopathological findings reported were defined using Amsterdam Consensus Criteria [8]. Histological lesions were accordingly classified as lesions due to maternal vascular underperfusion (MVU) or foetal vascular underperfusion (FVU).

For maternal and foetal inflammatory response, stage and grade were evaluated using Amsterdam consensus guideline [8] and immunohistochemical analysis with anti-CD3, anti-CD8, anti-CD20, anti-CD68 antibodies and CD15 was performed [9].

Moreover, CD15 immunoreactivity was also used as and as biomarker of foetal hypoxia in accordance of the study of Seidman et al. [10].

To quantify the number of Hofbauer cells, immunohistochemical analysis was performed using an anti-CD4 antibody [11]. All histologic findings were compared with those present in a placenta at 39 weeks, from a healthy pregnancy, with physiological course and spontaneous delivery, previously examined in our department (*case control*).

On histological examination, the case control showed villi with appropriate maturation for gestational age, normal development of vascular-syncytial membranes and this did not present lesions due to maternal or foetal malperfusion.

## Transmission Electron microscopy

For ultrastructural analysis, small fragments of placental tissue routinely fixed in 10% buffered formalin were washed with 0.1 M phosphate buffer, pH 7.2, and then fixed in Karnovsky solution (4% paraformaldehyde, 5% glutaraldehyde) for 3 h at room temperature. The samples were postfixed in 1% osmium tetroxide, dehydrated in graded acetone, and embedded in Durcupan-Araldite epoxy resin.

Semithin sections were stained with toluidine blue and examined by light microscopy to select representative sections. Ultrathin (80 nm thick) sections were cut using an LKB ultramicrotome, stained with Uranyless EM stain/lead citrate, and examined using a Philips-FEI EM.

## Clinical data for the pregnant women and newborns

The clinical data for the pregnant mothers are provided in Table 1. The considered cases referred to the first wave of COVID 19. They were enrolled in the period March–November 2020 when the new variants of SARS-CoV-2 were not yet widespread throughout our Italian Region (Emilia-Romagna) [12].

**Table 1:** Clinical data of pregnant women

	Case 1	Caso 2	Case 3	Case 4	Case 5
Age	36 yrs	35 yrs	32 yrs	26 yrs	30 yrs
Swab Positive	36 wks	32 wks	34 wks	33 wks	25 wks
CT	NA	NA	Bilateral interstitial pneumonia (visual score:20%)	Bilateral ground glass areas, suggestive for interstitial pneumonia (visual score 15%)	NA
Symptoms	Fever, Cough, Vomit, diarrhea, loss of smell, asthenia	Fever, Cough, malaise	Fever, acute respiratory distress syndrome	Fever, cough, dyspenea	Loss of smell, asthenia, malaise, cough
Maternal comorbidity	Hypothyroidism	Hypothyroidism	Gestational diabetes	NO	NO
Treatment	NO	NO	Non-invasive ventilation (CPAP)	NO	NO

*CPAP* Continuous Positive Airway Pressure, *CT* Computed Tomography, *NA* not avaiable, *wks* weeks, *yrs* years

The mothers’ ages ranged from 26 to 36 years. COVID-19 affected these women in the third trimester.

Case 5 involved earlier infection, at 25 weeks of gestation. For Case 3, the woman presented gestational diabetes and was treated with dietary therapy. Cases 1 and 2 were complicated by hypothyroidism. RT-PCR showed positivity for SARS-CoV-2 RNA in nasopharyngeal swabs from all patients. All of the pregnant women exhibited symptoms related to COVID-19, such as fever, cough, loss of smell, and malaise. Distress syndrome with fever and cough with dyspnoea, respectively, developed in Cases 3 and 4. Computed tomography for Cases 3 and 4 revealed signs of pneumonia with bilateral involvement, multiple lobular and subsegmental areas of consolidation and ground-glass opacities. The patient in Case 3 underwent non-invasive ventilation (continuous positive airway pressure) (CPAP). At the time of delivery, all of the women tested negative for SARS-CoV-2 by nasopharyngeal swab.

Table 2 shows the perinatal outcome of the newborns. All of the babies were born at term (range: 38–40 weeks). Birth weights were appropriate for the gestational ages, except in Case 5, which involved a slight reduction in neonatal growth [13]. The mode of delivery was spontaneous in four cases. In Case 3, the child was born by elective caesarean section upon maternal request.

**Table 2:** Clinical data of newborns

	Case 1	Caso 2	Case 3	Case 4	Case 5
Mode of delivery	SVD	SVD	ECS	SVD	VD
Gestational age (weeks)	40 + 4	38 + 5	38 + 2	39 + 6	39 + 4
Bith weight (gr)	3790	3535	2290	3720	2750
Head circumferences (cm)	35	36	35	36	34
Length (cm)	52	53	50	49	50
Apgar score 1′-5′	9; 9	9; 9	9; 10	9; 9	9; 10
Amniotic Fluid	Clear	Clear	Clear	Stained	Clear
Exclusuvely breastfeeding	Yes	Yes	Yes	Yes	Yes
Clinical signs or symptoms of infection	NO	NO	NO	NO	NO
Nasopharygeal swab for SARS CoV2	Negative	NA	Negative	Negative	NA

*ECS* Elective Cesarean Section, *NA* not avaiable, *SVD* spontaneous vaginal delivery

The Apgar score for all infants was normal. The amniotic fluid was clear in all cases, except in Case 4, in which the fluid was stained.

None of the newborns had signs or symptoms related to COVID-19. A nasopharyngeal swab for SARS-CoV-2 was performed in three cases, all with negative results.

All the newborns stayed with their mother (rooming-in), exclusively receiving breast milk, and were discharged on the 3rd day of life.

## Pathological findings

### Macroscopic, microscopic and immunohistochemical findings

Table 3 summarizes the macroscopic, microscopic and ultrastructural findings of five placentas.

**Table 3:** Pathological placental patterns

	***Case 1***	***Case 2***	***Case 3***	***Case 4***	***Case 5***
Placental weight,g	374	485	465	570	303
Expected weight for gestational age	^a^481 + 68	^a^452 + 58	^a^452 + 58	^a^452 + 58	^a^481 + 68
Neonatal weight g	3790	3535	2290	3720	2750
Expected weight for gestational age	^a^3222 + 439	^a^3001 + 475	^a^3001 + 475	^a^3001 + 475	^a^3222 + 439
***MVU lesions***					
Infarction	–	–	–	–	–
Increased privillous fibrin deposition	–	–	–	–	–
Accelerate villous maturation	–	–	–	–	–
Tunney-Parker Change	–	–	+	–	–
Distal villous hypoplasia	–	–	+		
Intervillous Thrombi	+ (1)	+ (2)	–	–	+ (5)
***FVU lesions***					
Thrombi in fetal circulation	–	–	–	–	–
Avascular villi	–	–	–	–	+ (2 foci)
Delayed villous maturation	+	+	–	+	–
Chorangiosis	–	+	–	+	+
Phagocytosis of meconium in the amniotic membranes	–	–	–	+	–
***INFLAMMATORY Patterns***					
Chorionamnionitis	–	+	–	–	–
Chronic Villitis	–	–	–	+	+
Presence of spherical like-coronavirus particles, on ultrastructural analysis	+	+	+	+	+

*MVU* Maternal vascular Underperfusion

*FVU* Foetal vascular Underperfusion

^a^By Gruenwald P, Minh H Am j Clin Pathol 1960; 34:247–253. doi: 10.1093/ajcp/34.3.247 [13]

from mothers affected by COVID-19. Lesions were classified as MVU or FVU and inflammatory changes.

Both placental weight and birth weight of the newborns were appropriate for the gestational age, except for Case 5, in which a mild reduction in neonatal growth and a mild reduction in placental weight (neonatal weight: 2750 g, expected weight: 3222 + 439 g; placental weight: 303 g, expected weight: 481 + 68) were found (Table 3) [13]. On macroscopic examination, the lesions observed in placental discs, as oval or round sharply delineated laminated haemorrhagic or yellow and white lesions on cut section in Cases 1, 2 and 5 (Table 3) corresponded histologically to intervillous thrombi. These lesions are an expression of MVU.

In Case 1, there was only one thrombus; in Cases 2 and 5, there were multiple lesions. In the Case 5 there were five lesions, with a large diameter ranging from 0.5 to 2 cm.

**Fig. 1:**
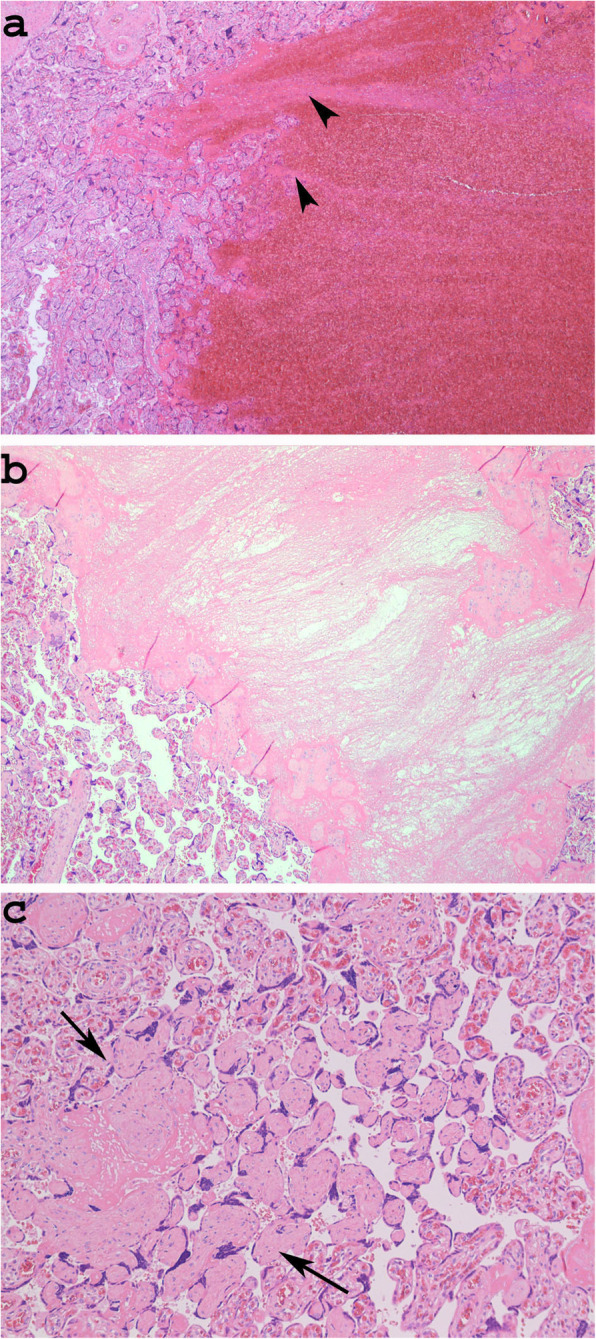
Histological section of a recent thrombus showing alternating layers of red blood cells and fibrin (**a** Case 5, Haematoxylin and eosin stain× 40, arrowheads: layers of fibrin). Note the absence of chorionic villi, which are displaced to the periphery. An old thrombus with uniform granular eosinophilic appearance (**b** haematoxylin and eosin stain × 40). Avascular villi with eosinophilic pauci-cellular stroma (**c** Case 5, haematoxylin and eosin stain × 100, Arrows: Avascular villi)

Histologically, recent thrombi showed alternating layers of red blood cells and fibrin; they typically contained no chorionic villi, which were displaced to the periphery (Fig. 1a). In contrast, older thrombi had a uniform granular eosinophilic appearance because as thrombi age, the layers of red blood cells degenerate (Fig. 1b).

Other macroscopically evident lesions found in Case 5 were two pale triangular areas histologically corresponding to an FVU lesion, known as avascular villi. This lesion was characterized by the presence of foci of distal villi that displayed eosinophilic pauci-cellular stroma. Each lesion contained more than 10 avascular villi and was thus considered a large focus (Fig. 1c) [14] (Table 3).

**Fig. 2:**
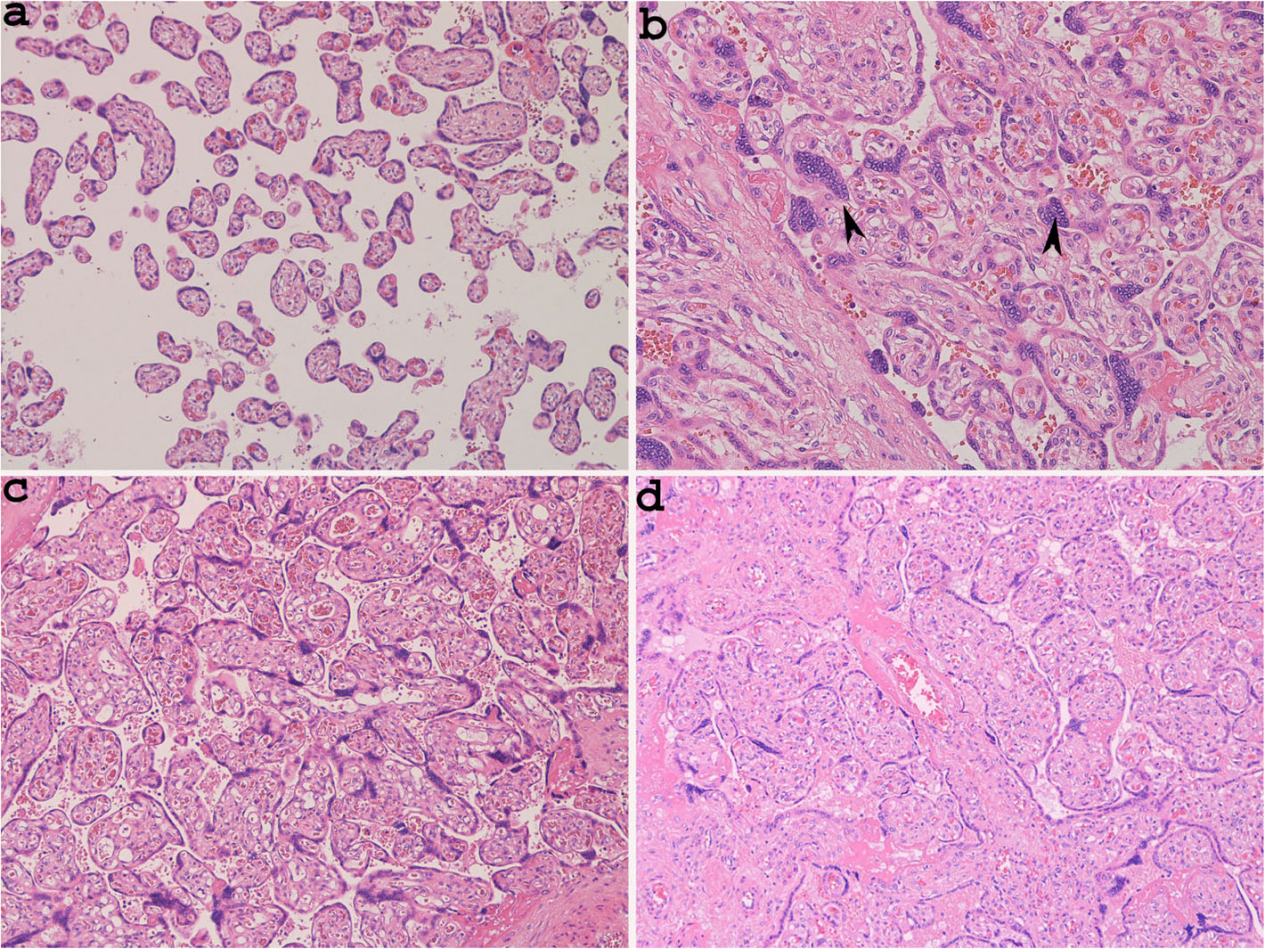
Distal villous hypoplasia with elongated and slender distal villi and wide intervillous spaces (**a** Case 3, haematoxylin and eosin stain × 100) and Tenney-Parker change, with villi showing an increased number of syncytial knots (**b** Case 3, haematoxylin and eosin stain × 100, arrowheads: syncytial knots). Chorangiosis with terminal villi containing numerous capillaries (**c** Case 2, haematoxylin and eosin stain, × 100); delayed maturation (**d** Case 1, haematoxylin and eosin stain × 100)

The MVU lesions detected only on histological examination involved distal villous hypoplasia (DVH) and Tenney-Parker change. Both lesions were observed in Case 3. DVH was found only in a small area of one slide and can be considered a placental villous maldevelopment [15]. This lesion was characterized by the presence of elongated and slender distal villi and a wide intervillous space (Fig. 2a). The Tenney-Parker change corresponded to an increased number of syncytial knots that appeared as multi-layered aggregates with at least 5 syncytiotrophoblast nuclei protruding from the villous surface (Fig. 2b).

All remaining lesions were only detected on microscopic examination and corresponded to lesions due to MVU or FVU or both.

The most frequently observed lesions relating to FVU corresponded to chorangiosis and delayed villous maturation. Chorangiosis was characterized by the presence of > 10 terminal villi, which in turn contained > 10 capillaries in more than 3 different areas (Cases 2, 4, 5) (Table 3) (Fig. 2c) [16].

Delayed villous maturation was observed in Cases 1, 2 and 4. This lesion was characterized by the.

presence of enlarged distal villi, with centrally located capillaries and reduced vasculo-syncytial membrane counts (Table 3) (Fig. 2d) [17].

Inflammatory changes were observed in Cases 2, 4 and 5.

**Fig. 3:**
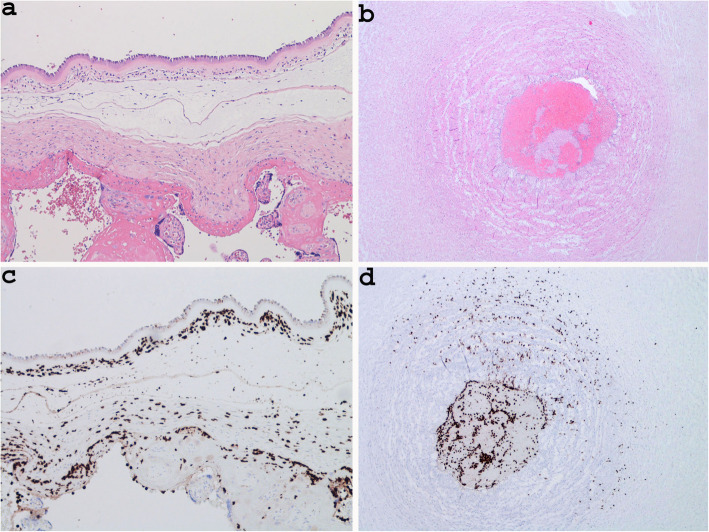
Histological section showing chorioamnionitis with patches of neutrophil granulocytes in the context of connective tissue located below the amniotic epithelium (**a** Case 2, haematoxylin and eosin stain × 200); indecipherable umbilical arteritis on haematoxylin and eosin stain in the same case (**b** haematoxylin and eosin stain × 40), the same sections on immunohistochemical analysis showing positivity for CD15 in the amniotic membrane (**c** × 100) and in smooth muscle of one umbilical artery and Wharton’s jelly (**d** × 40)

In Case 2, the inflammatory lesion was due to chorioamnionitis, which characteristically presented patches of neutrophil granulocytes in the context of the connective tissue lying below the amniotic epithelium (Stage 2; intermediate) (Fig. 3a) [8, 18]. To better quantify the inflammatory response in the umbilical cord and amniotic membranes we performed an immunohistochemical analysis, using CD15 antibody. In amniotic membrane inflammation and positivity to CD15 was observed in the chorion and amnion (stage 2, grade 1) according to Amsterdarm classification [8, 18] (Fig. 3a and Fig. 3c). In the umbilical sections stained with haematoxylin and eosin, the granulocytic infiltration was not evident (Fig. 3b). On contrary, immunohistochemical analysis showed immunoreactivity for CD15 in the smooth muscle of an umbilical artery and focally in the Wharton’s jelly, demonstrating presence of granulocytic inflammation (score 3 by Hatano et al. and stage 2 by Amsterdarm’s criteria) [8, 9] (Fig. 3d) (Table 4).

**Table 4:** Results of CD 15 immunoreactivity to evaluate inflammatory response and risk for intra-uterine foetal hypoxia

	***CASE 1***	***CASE 2***	***CASE 3***	***CASE 4***	***CASE 5***
Veins/Arteries of stem Villi	Absent/Absent	Focal/Absent (Low expression) ^a^	Focal/Absent (Low expression) ^a^	Absent/Absent	Absent/Absent
Capillaries of the terminal villi	Absent	Very rare < 50%^a^	Rare and focal (< 50%) ^a^	Many (< 50%)^a^	Rare and focal (< 50%)^a^
Umbilical vessels	Absent	Present in an umbilical artery involving both its smooth muscle and Wharton’s jelly (score 3)^b^ Amsterdarm’s stage 2	Absent	Absent	Absent
Amniotic membranes	Absent	Present involving fibrous chorion and amnion (stage 2) grade 1	Absent	Absent	Absent

^a^ Seidmann L et al. (ref [10])

^b^ Hatano et al. (ref. [9])

**Fig. 4:**
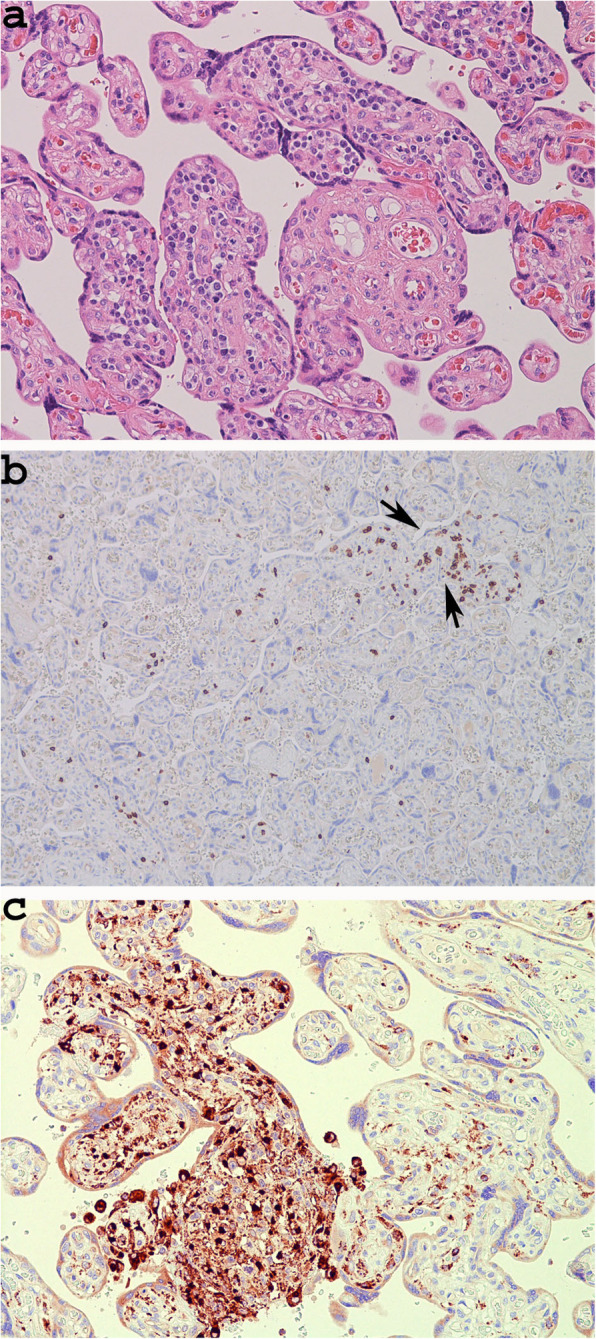
Chronic villitis (**a** Case 5, haematoxylin and eosin stain × 200); positivity for CD8 demonstrating the presence of T cytotoxic lymphocytes in the inflammatory infiltrate (**b** × 100, Arrows: villi with immunoractivity for CD8) and positivity for CD68 in the histiocytic component (**d** × 100)

In Cases 4 and 5, the inflammatory lesions were due to chronic villitis. The inflammation involved < 10 contiguous villi, and, as in both Cases 4 and 5, was found on only one slide, termed focal low-grade [19] (Fig. 4a).

According to immunohistochemical analysis, the inflammatory infiltrate of villitis revealed the presence of T cytotoxic lymphocytes positive for CD8 (Fig. 4b) and histiocytes positive for CD68 (Fig. 4c). We did not detect any cases positive for CD3 or CD20. Conversely, no case showed intervillositis with extensive inflammation in the intervillous space, and few histiocytes in the intervillous space positive for CD68, were only found in Case 5.

In Case 4, phagocytosis of meconium in the amniotic membranes consistent with acute intrauterine hypoxia during delivery was also observed.

In this case, as well as in all remainig cases, to better quantify the risk of hypoxia/asphyxia for newborn, we performed a immunohistochemical study, using CD15 immunoreactivity, in line with the study of Seidmann et al. [10].

**Fig. 5:**
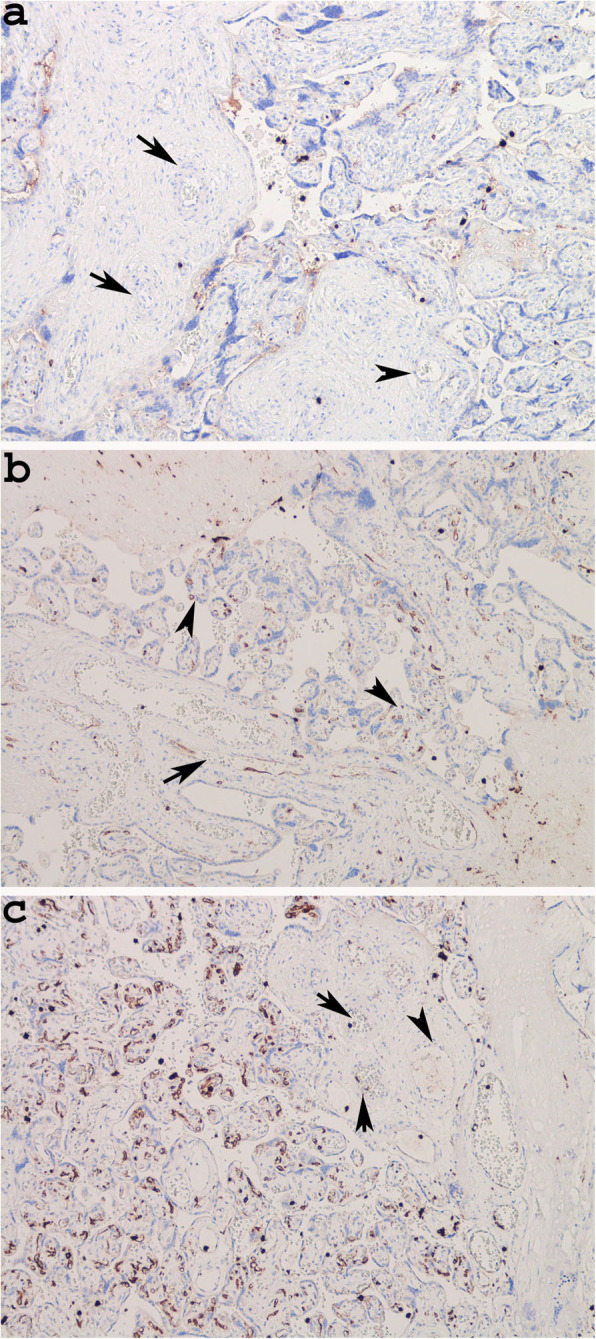
CD15 immunoreactivity in placental tissue. Negativity for CD15 in case 1, both in vessels of stem villi and capillaries of terminal villi (Case 1, **a** × 100, Arrows: arteries, Arrowhead: venous vessel); Focal positivity in a venous vessel of stem villi and in the a few capillaries of terminal villi (Case 3: **b** × 100; Arrow: Venous vessel, Arrowheads: positive capillaries); negativity for CD15 in vessels of stem villi and positivity in many capillaries, of a focus of terminal villi (Case 4: **c** × 100; Arrows: arteries, Arrowhead: venous vessel)

In the Case 1, we did not observe immunoreactivity for CD 15 in the arteries of the stem villi and in the capillaries of the terminal villi (Fig. 5a) (Table 4), as well as in the placenta of a healthy pregnancy (Case control), demonstrating that there was no risk of foetal hypoxia/asphyxia in this case of mild disease [10] (Table 1). In the remaining four cases, which corresponded to mild and more severe disease (Table 1), the expression of CD15 was low (Fig. 5b) or absent in the veins and arteries of stem villi, while in the capillaries of teminal villi was very low (Case 2), or involved small areas of placental tissue (cases: 3, 4, 5), which did not extend for more than 50% of a whole slide, at low magnification (Fig. 5c) (Table 4). Therefore in none of mild or more severe COVIDs we observed risk of severe foetal hypoxia/asphyxia and distal vascular immaturity (Table 4) (Fig. 5) [10].

**Fig. 6:**
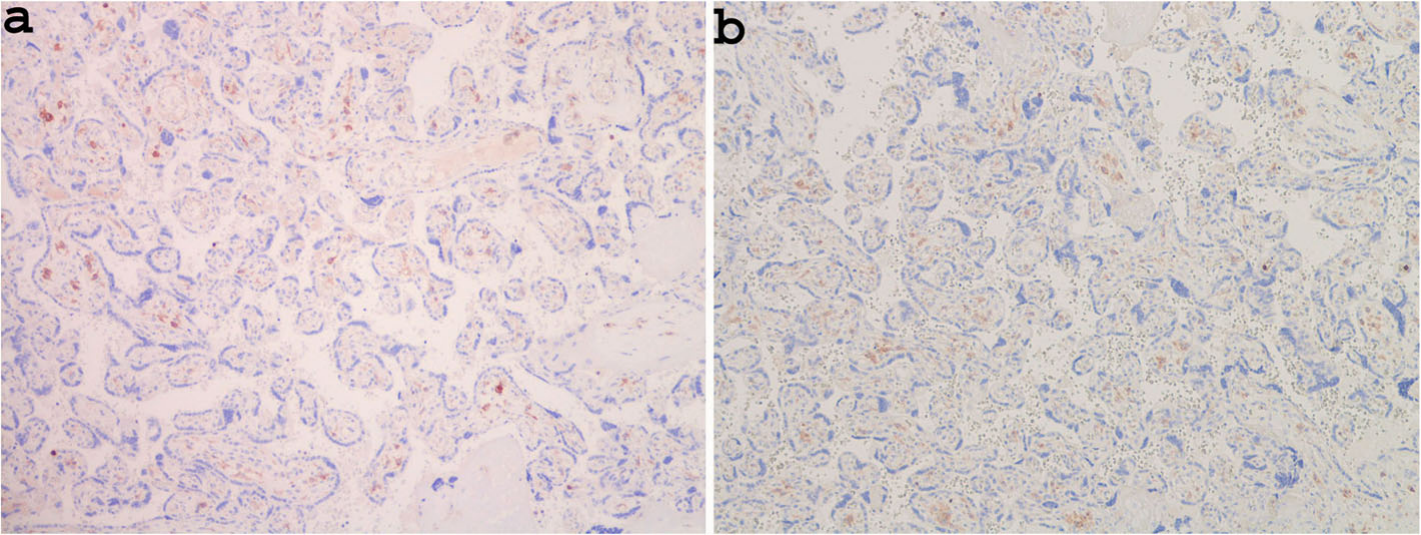
Immunoreactivity for CD4 in the case control sample from a normal pregnancy, revealing the number of Hofbauer cells (**a** × 100), which is the same as present in cases from a COVID-19-positive woman (Case 3, **b**: × 100)

Immunohistochemical analysis for CD4 revealed the presence of Hofbauer cells within the stroma of villi that did not show an alteration in their amount compared with that observed in the placenta of a healthy pregnancy (Figs. 6a and b).

### Ultrastructural analysis

**Fig. 7:**
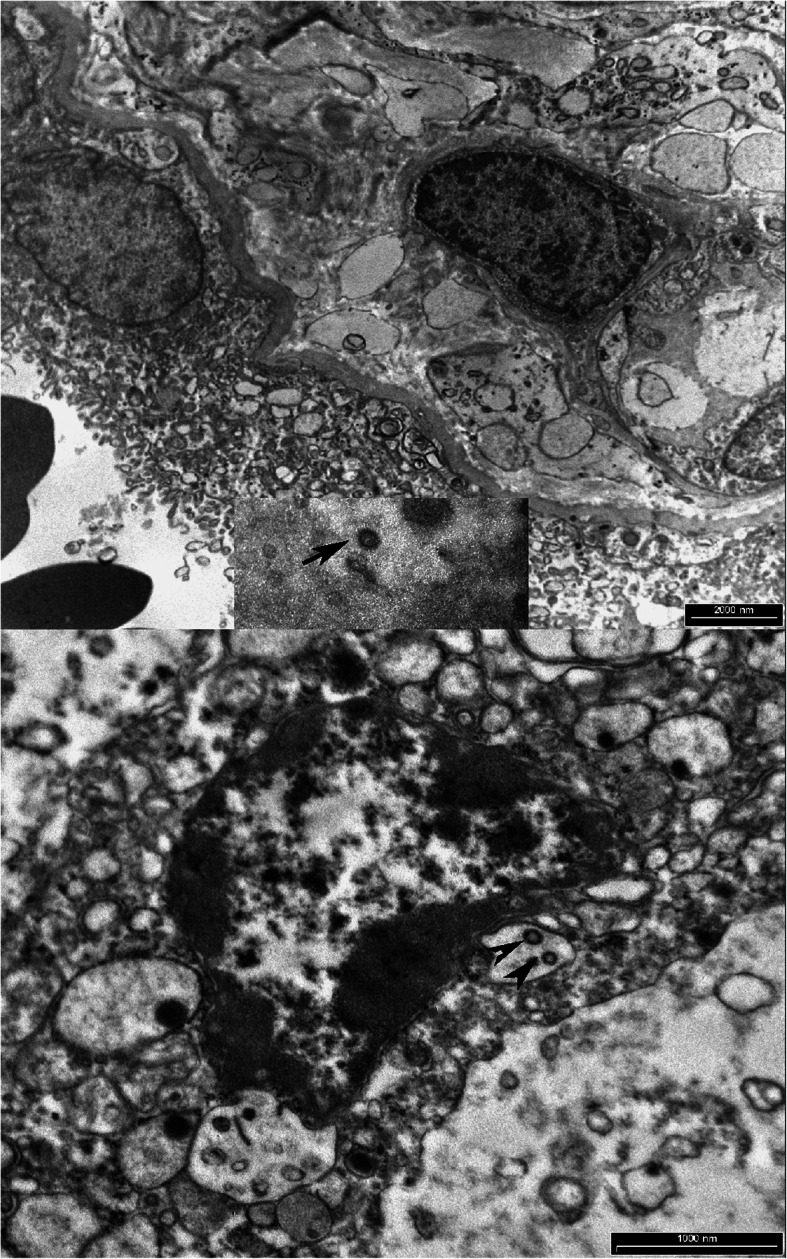
Ultrathin sections showing spherical-like coronavirus particles free in the cytoplasm of syncytiotrophoblasts (**a**: Case 3, Arrow: intracellular spherical-like coronavirus particle) or within a vesicle of syncytiotrophoblasts (**b**: Case 5, arrowheads)

In all cases, spherical-like coronavirus particles were identified in ultrathin sections as structures with an electron intermediate-density core and spike-like structures protruding from their surface. These virus-like particles were free in the cytoplasm (Fig. 7a) or within the cytoplasmic vesicles of syncytiotrophoblasts (Fig. 7b).

### Follow-up

Currently, both the mothers and infants of this cohort are alive and well.

## Discussion

During the COVID-19 pandemic, the role of the placenta as a barrier against SARS-CoV-2 infection was hypothesized. The placenta is a fundamental and enigmatic organ; it is vital for the development of the foetus to its full potential, and any changes in placental function can modify the course of pregnancy and impact the health from birth to childhood. The placenta often has a protective role, inhibiting the transmission of infectious agents from the mother to the foetus because of the presence of the trophoblastic epithelial lining on the surface of chorionic villi, in addition to the presence of macrophages in both the villous stroma (Hofbauer cells) (HCs) and in the decidua. Nonetheless, many infectious agents are able to pass through the placenta [20, 21], leading to foetal abnormalities (e.g., microcephaly in Zika virus infection) [22] or direct placental damage (e.g., villitis from cytomegalovirus) [23], which may cause foetal growth restriction (FGR) or demise.

The role of the placenta as a barrier preventing the transmission of SARS-CoV-2 to the foetus remains unresolved.

Many studies have suggested that the entry and spread of the virus within human cells is favoured by angiotensin-converting enzyme (ACE) inhibitors and angiotensin II receptor blockers (ARBs), which increase expression of ACE2 receptors. These receptors are ubiquitous, particularly abundant in the lung, but also expressed in the reproductive organs, placenta, uterus and maternal-foetal interface during pregnancy [24–27]. Moreover, ACE2 receptors are highly expressed in foetal tissues such as the heart and liver [26].

Thus, ACE2 receptors might facilitate COVID-19 transmission from the mother to the developing foetus. Nevertheless, many authors have reported no case of definitive vertical transmission to an infant of a woman with COVID-19 [28–31]. Conversely, other studies have suggested that SARS-CoV-2 may cause death in the first and second trimesters of gestation. Unfortunately, in some cases of miscarriage, there was no placental examination or testing for the presence of SARS-CoV-2 in the placenta or foetus [32, 33]. In other study histological placental examinations have shown evidence of inflammation [34] or diffuse perivillous fibrin, infarcts and the presence of both macrophages and T cells [7, 35].

Vivanti et al. reported a proven case of transplacental transmission of SARS-CoV-2 to a newborn from a mother with COVID-19 during late pregnancy, that developed brain injury [7].

In this case, placental histological examination revealed diffuse perivillous fibrin deposition with infarction and acute and chronic intervillositis. In addition, an immunohistochemical study of placental tissue with antibodies against the SARS-CoV-2 N protein revealed intense cytoplasmic positivity of villous trophoblastic cells [7]. Moreover, Sisman et al. reported a preterm SARS-CoV-2 positive infant who developed severe acute respiratory syndrome, with immunohistochemical and ultrastructural findings in placental tissue consistent with SARS-CoV-2 infection [36].

Additionally, Facchetti et al. observed evidence of vertical transmission to a newborn who developed and interstitial pneumonia on chest X-ray in one case of 14 placentas from COVID-19-positive pregnant women [37]. Histological findings for the placental tissue showed histiocytic-neutrophilic intervillositis, which was absent in the remaining 13 cases. Immunohistochemical analysis of the placenta showed strong expression of the SARS-CoV-2 S and N proteins, and viral particles morphologically consistent with a coronavirus were observed in villous syncytiotrophoblasts, endothelial cells, fibroblasts, maternal macrophages and Hofbauer cells by ultrastructural analysis [37]. In contrast, Hsu et al. reported the case of a newborn delivered in the third trimester by a woman with mild COVID-19, with an uneventful course, COVID-19 RT-PCR test negativity, and placental histology findings consistent with MVU lesions. The immunohistochemical analysis using SARS-CoV-2 nucleocapsid-specific monoclonal antibodies demonstrated the presence of SARS-CoV-2 [38].

In our series, all newborns exhibited good health at birth, without COVID infection-related symptoms and histological examinations of the placentae showed MVU and FVU.

According to the literature, these lesions are common in the placenta of COVID-19-positive women [7, 38–49] in the third trimester, and might be related to hypoxia. In cases of COVID-19 in pregnancy, hypoxia in the third trimester may be related to severe disease, particularly affecting the respiratory system [50], but may also be due to more common physiologically adaptive changes occurring in pregnancy, such as: - increased blood volume and oxygen consumption, increased uterine volum [51]; − systemic inflammatory or hypercoagulable state [7, 40, 52, 53].

In our series of cases, the most common lesions on macroscopic examination were intervillous thrombi (Cases 1, 2 and 5).

Although intervillous thrombi are generally incidental findings [54], they may be associated with maternal hypertensive disorder [55–57] or diabetes [58] and coexist with infarctions; they may also be responsible for intrauterine growth retardation [59]. These lesions are frequent in cases of COVID-19 and may be related to hypercoagulability in the intervillous space [40, 53].

FVU-related lesions, such as avascular villi, are considered the consequence of an obstruction of large vessels in the placenta and are due to hypercoagulation, cardiac dysfunction, hypoxia, long hyper-coiled cords and abnormal umbilical cord marginal insertion [60, 61]. When avascular villi are extensive, a poor perinatal outcome, foetal demise, and neurodevelopmental sequelae can occur [62]. In our series, 10 avascular villi were observed in one case (Table 3). Nonetheless, this lesion was not associated with any gross abnormalities of the cord and its vessels or thrombi in the larger vessels of the chorionic plate. The perinatal outcome of the newborn was good.

Inflammatory lesions were observed in three cases: chorioamnionitis or chronic villitis. Chorioamnionitis has already been reported in pregnant women with SARS CoV-2 infection [41–43], and it may be considered an ascending uterine infection, which is probably not related to haematogenous infection due to SARS CoV-2. Chronic villitis of unknown aetiology, common in the third trimester of gestation, is considered an aberrant maternal response to antigens and may be associated with intra-uterine growth restriction, prematurity, and, in some instances, recurrent pregnancy loss [19].

In our cohort, unlike in cases reported by other authors [43], chronic villitis produced exceedingly small lesions, affecting only a small number of villi (< 10 contiguous villi). The chronic villitis was not associated with growth restriction. Mild growth restriction was observed in a case in which chronic villitis was associated with other FVU and MVU lesions, such as avascular villi and multiple intervillous thrombi.

Chorangiosis represented another frequent lesion observed by histological examination, consistent with literature [40, 60]. Chorangiosis represents an adaptative response to chronic underperfusion/hypoxia and is associated with decreased maternal oxygen saturation [63–65]. More frequently, this lesion can be found in gestational diabetes [66] and in cases of severe maternal anaemia, hypothyroidism [67], and preeclampsia [68].

The delayed villous maturation or distal villous immaturity observed in Cases 1, 2 and 4 has also been reported in the placenta from COVID-19-positive pregnant women [40].

Distal villous hypoplasia, a lesion due to MVU, was found in mother with severe COVID-19, pneumonia and respiratory distress. This lesion can be can be considered an attempt to adapt to maternal malperfusion, with an increase in the vasculo-syncytial membranes at the expense of further villus branching and angiogenesis. We also detected in the same case a Tenney-Parker change, another MVU lesion that represents an abnormality of the trophoblast, which forms an excessive number of syncytial knots in response to hypoxia and represents a common finding in the placenta of women with COVID-19 [47, 49].

Overall, a diagnosis of an excessive number of syncytial knots should be made with caution, considering there is a significant positive correlation of gestational age, reflecting placental maturity [69].

To quantify the risk of hypoxia/asphyxia for newborn, we performed an immunohistochemical study, using CD15 immunoreactivity, in line with the study of Seidmann et al. [10].

This marker is useful to evaluate granulocytic inflammation in the umbilical cord [9] as well as in the endothelial cells of placental tissue can be indicate distal vascular immaturity and risk of foetal hypoxia/asphyxia [10]. The immunoreactivity for this antibody in our study demonstrated that there was not severe foetal hypoxia/asphyxia risk for newborns or distal vascular immaturity. In fact, the expression of this marker was low or absent in the veins and absent in the arteries of the stem villi. In addition, in the capillaries of terminal villi its expression involved small areas of placental tissue in one slide at low magnification (< 50%) [10].

The observed numerical alteration in Hofbauer cells appears and interesting finding, as they seems to play an important role in the vertical transmission of some infectious agents during pregnancy.

Hofbauer cells are macrophages located within the foetal villi at an early stage of development [70]. The main functions of these cells include phagocytosis of fluids and apoptotic materials and antigen presentation in response to infectious agents [70, 71].

Moreover, they produce vascular endothelial growth factor (VEGF), a potent angiogenic growth factor, suggesting possible involvement in the regulation of angiogenesis in placental villi [72]. Additionally, it has been suggested that these cells participate in intrauterine transplacental transmission of certain infectious agents [73].

Unlike in some other reports [37, 53], we did not find a numerical increase in these cells.

On ultrathin sections, spherical-like coronavirus particles, with spike-like structures protruding from their surface, were found in the cytoplasm of syncytiotrophoblast cells. These subcellular structures correspond to those observed in most of the previous studies [35, 36, 46], in which they were inaccurately misinterpreted as viral particles [74]. More recently, Bullock et al. emphasized that intracellular coronavirus particles are typically located within membrane vacuoles and vesicles, that they are not found free within the cytoplasm and that their spikes are not in direct contact with the cytosol [74]. Furthermore, in cross-sections, the interior helical nucleocapsids of coronaviruses characteristically appear as electron-dense black dots [74], which was not observed in our cases or in many cases reported by other authors [35, 36, 46]. The lack of such findings indicates that these subcellular particles are not coronaviruses but rather correspond to other subcellular structures that mimic coronaviruses [74].

## Conclusion

Placental patterns in pregnancy affected by COVID-19 in late stages showed no evidence of vertical trans-placental SARS-CoV-2 transmission and with no significant impact on the perinatal outcome of the newborn in both mild and more severe disease. The majority of placental lesions are represented by maternal and/or fetal vascular malperfusion lesions.

### Strengths and limitations

This was a case reports study and it cannot exclude the possibility of a chance association. The selection of patients whose care makes up most case reports is subject to selection bias and may represent outliers in clinical practice, necessitating caution regarding the generalizability of results.

However, this paper summarizes the placental pathological presentations and perinatal outcome of cases with maternal COVID-19 infection. They represent first precious data for the further studies and clinical treatment.

### Perspective

Currently, both the mothers and infants of this cohort are alive and well. Thus, in our opinion, the negative impact of COVID-19 in pregnancy probably depends on multiple factors, such as the stage of gestation at the time of infection, genetic factors of the mother, or different genetic variants of the virus.

Given the numerous links between alterations of the placenta and neonatal disease, there is growing interest in understanding the complexity of this fascinating organ in COVID-19, which might impact the immediate prognosis of newborns and the risk of diseases post-natally. Focusing attention on the placenta, with more insight into cellular and molecular mediators associated with pregnancy complications, appears to be essential for the development of successful intervention and prevention strategies during pandemics.

## Data Availability

All data generated or analysed during this study are included in this published article.

## Abbreviations

ACE: Angiotensin-Converting Enzyme
COVID-19: Coronavirus Disease 19
CPAP: Continuous Positive Airwas Pressure
CT: Computed Tomography
FGR: Foetal Growth Restriction
FVU: Foetal Vascular Underperfusion
HC: Hofbauer Cell
H&E: haematoxylin and eosin
NA: Not Avaiable
MVU: Maternal Vascular Underperfusion
PHEIC: Public Health Emergency of International Concern
RT-PCR: Reverse transcriptase-polymerase chain reaction
SARS-CoV-2: Severe Acute Respiratory Syndrome Coronavirus 2
TORCH: Toxoplasmosis, Rubella, Cytomegalovirus, Herpes simplex
VEGF: Vascular Endothelial Growth Factor
WHO: World Health Organization
Wks: Weeks
yrs.: years
